# GABAergic Signaling Underlying REM Sleep Deprivation‐Induced Spatial Working Memory Deficits

**DOI:** 10.1002/brb3.70607

**Published:** 2025-06-02

**Authors:** Peeraporn Varinthra, Shu‐Ching Shih, Ingrid Y Liu

**Affiliations:** ^1^ Institute of Medical Sciences, College of Medicine Tzu Chi University Hualien Taiwan; ^2^ Department of Molecular Biology and Human Genetics Tzu Chi University Hualien Taiwan

**Keywords:** Alzheimer's disease, GABA, rapid eye movement, sleep deprivation, working memory

## Abstract

**Introduction:**

Declining spatial working memory (WM) is an early hallmark of Alzheimer's disease (AD). Sleep disturbance exacerbates spatial WM and increases AD risk. The GABAergic system, crucial for sleep regulation, may mediate this link. We thus investigate the relationship between spatial WM and hippocampal GABAergic signaling during rapid eye movement sleep deprivation (REM‐SD) in AD model mice.

**Methods:**

We assessed spatial and non‐spatial WM, locomotor activity, and anxiety‐like behavior in 6‐month‐old triple transgenic (3xTg) AD mice and wild‐type (WT) controls, with and without REM‐SD (5 days, 4 h/day). We then used immunofluorescence to quantify GABA_A_α1, GABA_B_R1, GAD67, and GABA levels in the prefrontal cortex (PFC) and hippocampus and analyze the correlations with behavioral outcomes.

**Results:**

REM‐SD increased locomotor activity, reduced anxiety‐like behavior, and improved non‐spatial WM in 3xTg‐AD mice. Conversely, REM‐SD impaired spatial WM in WT mice, which was also demonstrated in 3xTg‐AD mice. Increased hippocampal GABA levels are correlated with improved non‐spatial WM in 3xTg+SD mice. In contrast, impaired spatial WM in WT+SD mice was associated with elevated hippocampal GABA and GABA_B_R1, decreased hippocampal GAD67, and reduced PFC GABA levels. Notably, spatial WM in 3xTg+SD and 3xTg control mice related to increased GABA_A_α1 in the PFC and hippocampus and GAD67 in hippocampal CA1, along with decreased GABA_B_R1 and GAD67 in the dentate gyrus.

**Conclusion:**

REM‐SD‐induced alterations in WM performance are linked to GABAergic signaling changes in the PFC and hippocampus, with distinct patterns in WT and 3xTg‐AD mice. This study provides insight into AD pathologies and potential therapeutic targets for sleep‐related cognitive impairments.

## Graphical abstract

Rapid eye movement (REM) sleep deprivation (SD) has been shown to impair working memory performance, potentially through disruptions in GABAergic signaling within the prefrontal cortex (PFC) and hippocampus. These alterations exhibit distinct patterns between wild‐type (WT) and 3xTg‐AD mouse models.

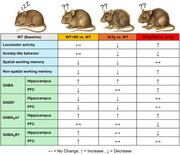

## 1 Introduction

Sleep is a fundamental physiological process essential for maintaining both physical and mental well‐being. Optimal sleep quality supports immune function (Besedovsky et al. 2012), tissue repair, emotional regulation (Krueger et al. 2016; Van Cauter and Plat 1996; Vandekerckhove and Wang 2018), learning, and memory consolidation (Feld and Diekelmann 2015). The National Institute of Health of the USA defines sleep deprivation (SD) as insufficient sleep duration or poor sleep quality each night (Abrams 2015). SD arises from various factors, including prolonged work hours (Costa 2015) and stress‐induced allostasis disruption or allostatic overload (McEwen and Karatsoreos 2015). Acute SD impairs cognitive functions such as attention and decision‐making, as well as working memory (WM) (Banks and Dinges 2007; Durmer and Dinges 2005; Goel et al. 2013), and affects mood and judgment (Pires et al. 2016), while chronic SD elevates accident rates (Barger et al. 2006) and mortality risks (Cappuccio et al. 2010; Gallicchio and Kalesan 2009; Kurina et al. 2013; Y. Wang et al. 2021). Notably, shortened sleep duration and increased daytime sleepiness in middle‐aged and elderly individuals correlate with a higher incidence of Parkinson's disease (Rosinvil et al. 2024), stroke (Tsai et al. 2022), and dementia (Li et al. 2023; Lv et al. 2022; Rosinvil et al. 2024; Sabia et al. 2021), suggesting a critical link between sleep and neurodegenerative diseases.

Alzheimer's disease (AD) is the most common cause of dementia (Burns and Iliffe 2009) and affects millions of people worldwide, with prevalence continuously increasing (Prince et al. 2013). Sleep disturbances are highly prevalent in AD patients (Van Erum et al. 2018), and SD can exacerbate AD pathology. Specifically, SD may promote the accumulation of amyloid‐beta (Aβ) plaques and the formation of neurofibrillary tangles (NFT) (Clark et al. 2015b), which are two key pathological hallmarks of AD. Deposition of Aβ plaques and increase of NFT disrupt neuronal transmission, glial activation, and immune responses (Rajmohan and Reddy 2017), ultimately leading to synaptic dysfunction and cognitive decline.

Rapid eye movement (REM) is a crucial stage of the sleep cycle (Marks et al. 1995; Siegel 2011) and plays a vital role in consolidating spatial (Boyce et al. 2017), working (Lau et al. 2015), and emotional memories (Ai and Dai 2018). The meta‐analysis study reported that reduced REM sleep correlates with cognitive impairment in AD patients (Zhang et al. 2022). Additionally, Chen L et al. have shown that REM‐SD increased Aβ accumulation and cognitive deficits in rats (Chen et al. 2017). Therefore, understanding the impact of REM‐SD on AD‐related cognitive decline and the underlying mechanism is critical for prevention.

The GABAergic system is a primary inhibitory neurotransmitter system known to regulate both sleep (Gottesmann 2002) and WM (Marsman et al. 2017; Ragland et al. 2020), particularly through activation of GABA_A_ and GABA_B_ receptors (Hirouchi and Kuriyama 1994). Dysfunctional GABA_A_ and GABA_B_ receptor signaling have been implicated in defective memory consolidation in AD models (Calvo‐Flores Guzman et al. 2018). The study examined individuals aged eighteen to sixty‐five and discovered that reduced levels of GABA_A_R α1 and α2 subunits mRNA in the peripheral blood correlated with a higher incidence of insomnia (Xiang et al. 2023). It was also noted that the GABA_B_R1 receptor expression is elevated in the hippocampus CA1 area of mice experiencing SD (Tadavarty et al. 2011). Even though many studies show a link between REM‐SD and GABAergic signaling, it is still unclear which brain areas are relevant to WM and how they affect AD.

Therefore, in the present study, we aim to investigate the relationship between REM‐SD, GABAergic signaling‐related molecular expressions (GABA_A_α1, GABA_B_R1, glutamate decarboxylase 67 (GAD67), and GABA), and WM performance in the PFC and hippocampal regions of six‐month‐old WT and 3xTg‐AD mice.

## 2 Materials and Methods

### 2.1 Animals and Ethics Statement

This study used six‐month‐old B6129SF1/J and 3xTg‐AD mice. The 129 mice were purchased from BioLASCO Taiwan Co. Ltd. and bred with C57BL/6 mice at the Tzu Chi Laboratory Animal Center in Hualien, Taiwan, to generate the B6129SF1/J mice. The 3xTg‐AD mice were initially bought from Jackson Laboratory (stock no. 34830 JAX) and contained three mutation genes related to familial AD, including APP Swedish, PSEN1 M146V, and MAPT P301L. All mice were maintained in a temperature‐controlled room (12 h light/dark cycle) in the Laboratory of Animal Center of Tzu Chi University, Hualien, Taiwan. Food and water were freely accessed ad libitum. All the experimental protocols were reviewed and approved by the Institutional Animal Care and Use Committee of Tzu Chi University (IACUC no. 111084 and 112058).

### 2.2 Experimental Procedures

Mice were divided into four groups: (1) wild‐type (WT), (2) WT+SD, (3) 3xTg, and (4) 3xTg+SD. From day 1 to day 5, mice were induced REM‐SD using the modified multiple‐platform method. On day 6, mice were tested for spatial WM using the T‐maze test, and locomotor and anxiety‐like behavior were assessed using an open field test (OFT). From day 7 to day 8, the novel object recognition (NOR) test was used to measure the non‐spatial WM of mice. After that, all mice were terminated by transcardial perfusion for brain harvesting.

### 2.3 The Modified Multiple‐platform Method for Inducing REM‐SD

The modified multiple‐platform method induced REM‐SD in animal models and influenced memory impairment (Siddique et al. 2018). This study used a plastic cage measuring 41×34×16.5 cm (l x b x h) and having fixed platforms (2.5 cm in diameter and 3.5 cm in height) filled with water up to 1 cm beneath the platform surface. Mice were put on the platforms for 4 h daily for five consecutive days (Azogu et al. 2015) (8:00 AM‐12:00 PM). When the mice fell asleep, they dropped into the water, woke up, and climbed onto the platform.

### 2.4 Open Field Test

OFT is used to assess mice's locomotor activity and anxiety‐like behavior (Suresh et al. 2022). The white floor chamber size of 50×50×50 cm (l × b × h) was utilized and divided into inner and outer zones. Initially, the mice were placed in one corner of the chamber and allowed to investigate for 10 min. The traveled distance, speed, and time spent by mice in each zone were recorded and analyzed by video tracking software (EthoVision XT 17, Noldus Information Technology, Wageningen, Netherlands).

### 2.5 T‐maze

The T‐maze test was performed to measure the WM performance of mice (Suresh et al. 2022). The T‐shaped chamber, with a white wall and floor, was divided into the right arm (familiar arm), left arm (novel arm), middle zone, and starting arm. The movable wall divided the middle zone into left and right sides, and two cues were placed on the chamber wall toward the starting arm to help guide the mice walking to each arm. In the training trial, the moveable wall blocked the left arm, and then mice were placed on the starting arm to explore only the right arm for 5 min. Subsequently, the mice were put back in the holding cage for 2 min. In the testing trial, the movable wall at the left arm was removed, and again, mice were placed on the starting arm to observe the whole maze for 5 min. The time spent in both arms, the frequency of visiting the novel arm, and the alternative percentage in the novel arm were recorded and analyzed by video tracking software (EthoVision XT 17, Noldus Information Technology, Wageningen, Netherlands). The alternative percentage was determined by the number of visits to the novel arm/ (the number of visits to the novel arm + the number of visits to the familiar arm) ×100. Mice with typical WM performance would allocate similar or more significant amounts of time in the novel arm. In contrast, poor WM mice would spend more time in the familiar arm.

### 2.6 Novel Object Recognition Test

NOR was used to investigate non‐spatial WM in mice following the previous study protocol (Lueptow 2017). The test was performed in the size 50×50×50 cm (l × b × h) chamber for three days. For the first two days, the mice were allowed to explore the chamber for habituation without objects for 10 min each trial. On the third day, two objects of the same shape and size were introduced into the chamber. A training trial was performed first, in which the objects were placed diagonally to each other. Next, the mice were allowed to explore them for 5 min, followed by a recovery period of 5 min in the home cage. For the testing trial, one of the objects was replaced with a novel object, and mice again explored the object for 5 min. The video tracking software (EthoVision XT 15, Noldus Information Technology, Wageningen, Netherlands) was used to analyze the results of the time observed for each object and exploration duration. The discrimination index was determined by (time observed for a novel object—time observed for a familiar object)/(time observed for a novel object + time observed for a familiar object). Poor memory mice would spend less time observing a novel object than a familiar one.

### 2.7 Immunofluorescent Staining and Image Analysis

The brain tissues were harvested by anesthetizing mice with isoflurane and transcardial perfusion. The 0.9% saline was perfused through the hearts of mice, followed by 4% paraformaldehyde (PFA). The brain tissues were extracted, put in 4% PFA overnight, and transferred to 30% sucrose. After the brain sank, the brain sectioning processes were performed to get a 30 µm thickness of a coronal section. Sections were then fixed with methanol for 5 min at 4°C and blocked for 1 h at room temperature using 1% normal goat serum in 1X phosphate buffer saline (PBS), including 0.3% Triton X‐100. Then, sections were incubated overnight at 4°C with primary antibodies, including rabbit anti‐GABA_A_α1 (1:200, ab33299, Abcam), rabbit anti‐GABA_B_R1 (1:200, ab238130, Abcam), rabbit anti‐GAD67 (1:200, ab213508, Abcam), and rabbit anti‐GABA (1:200, GTX125988, GeneTex). The washing buffer, consisting of 1X PBS with 0.25% Triton X‐100, was used to wash sections three times, ten minutes per time. DAPI was used to counterstain the nucleus for ten min, and sections were rinsed with a washing buffer. Subsequently, sections were mounted on a slide by Fluoromount^TM^ aqueous mounting medium (F4680, Sigma‐Aldrich). The interested protein expressions were observed under a Nikon C2si+ confocal microscope (Nikon, Tokyo, Japan) and quantitated using ImageJ (downloaded from National Institutes of Health, Bethesda, MD, USA, https://imagej.net/ij/). Three fields (200×200 µm) per section of each area from three mice per group were quantified to calculate the fluorescence intensity of each antibody.

### 2.8 Statistical Analysis

All the graphs were plotted by GraphPad Prism 9.0 software (San Diego, CA, USA) as mean± standard error of the mean (SEM). The statistical difference at *p* < 0.05 was calculated using IBM SPSS 20. The effect of genotype and SD induction on locomotor activity, anxiety‐like behaviors, the alternation percentages in the T‐maze test, the total exploration time and discrimination index in the NOR test, and immunofluorescence staining were analyzed by two‐way ANOVA followed by the Mann–Whitney *U* test. The dependent samples T‐test was used to evaluate the time spent by mice in a novel arm and a familiar arm of the T‐maze. The correlation between behavior performances and GABAergic signaling in each brain area was calculated using Pearson's correlation coefficient analysis.

## 3 Results

### 3.1 SD Impairs Spatial WM of WT but Does Not Deteriorate WM Deficit in 3xTg‐AD Mice

**FIGURE 1:**
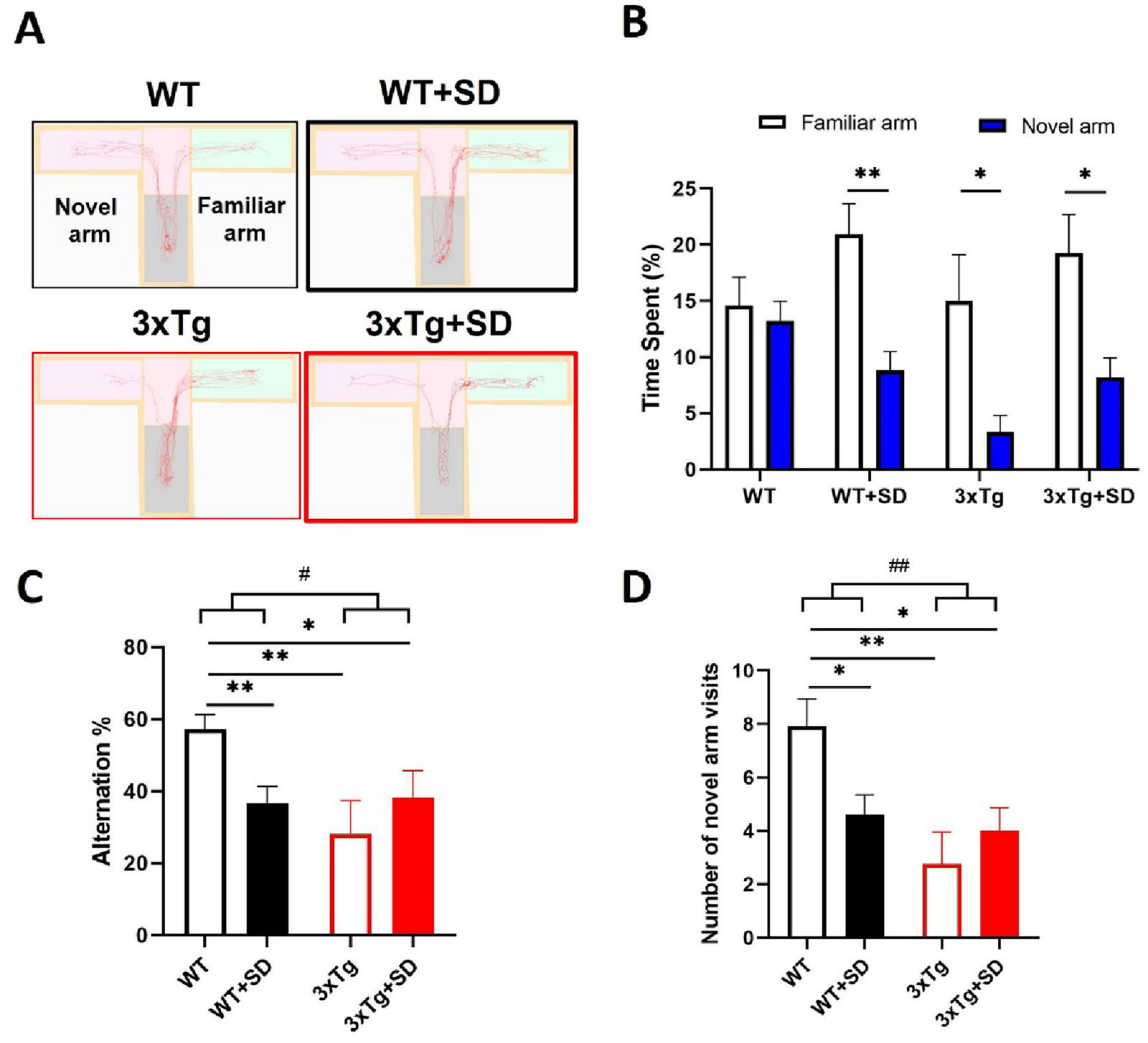
**SD impairs the spatial working memory of WT but does not deteriorate WM deficit in 3×Tg‐AD mice. (A)** The tracing diagram of mice in the T‐maze test lasts for 5 min, **(B)** The time spent by mice in familiar and novel arms, **(C)** The alternation percentage toward the novel arm, and **(D)** The number of novel arm visits by mice. All the results were plotted as Mean±SEM. The statistical differences were calculated for the interaction of two factors (genotypes and SD induction) by two‐way ANOVA and among groups by the Mann–Whitney *U* test. The paired sample t‐test evaluated significant differences within the group for (B). *Indicated *p*‐value < 0.05 and **Indicated *p*‐value < 0.01 when compared among groups and within groups. ^#^Indicated *p*‐value < 0.05 and ^##^Indicated *p*‐value < 0.01 when compared between genotypes. WT (*n* = 13), WT+SD (*n* = 10), 3xTg (*n* = 8), 3×Tg+SD (*n* = 9).

To study the effect of SD on spatial WM, we performed a T‐maze test. As shown in Figure 1A, the tracing diagram of mice in the T‐maze chamber consists of the familiar arm (right) and the novel arm (left) for 5 min. Comparing the time spent by mice in the familiar and novel arms (Figure 1B), the WT mice have no significant differences (*t*_(12)_ = ‐0.394, *p* = 0.701). On the contrary, the WT+SD (*t*_(9)_ = ‐4.067, *p* < 0.003), 3xTg (*t*_(7)_ = ‐2.445, *p* < 0.044), and 3×Tg+SD (*t*_(8)_ = ‐2.607, *p* < 0.031) mice significantly spent more time in the familiar arm than the novel arm. We noted a statistical difference in the interaction between the genotype and the SD induction on the alternative percentages (*F*_(3, 40)_ = 6.101, *p* < 0.018) and the visiting number to the novel arm (*F*_(3, 40)_ = 5.256, *p* < 0.028). The alternative percentages (Figure 1C) were significantly decreased in the WT+SD (*p* < 0.002), 3xTg (*p* < 0.01), and 3xTg+SD (*p* < 0.043) mice compared to WT mice. Besides, the number of visits to the novel arm (Figure 1D) by WT+SD (*p* < 0.049), 3xTg (*p* < 0.003), and 3xTg+SD (*p* < 0.017) mice were also significantly lower than that of the WT mice. However, the alternative percentages (*p* = 0.423) and the visiting number to the novel arm (*p* = 0.200) of 3xTg+SD mice were similar compared to 3xTg mice. These results indicated that SD impaired spatial WM in WT mice, like the symptoms of the 3xTg‐AD mice. SD induction did not increase the severity of spatial WM deficits in the 3xTg‐AD mice.

### 3.2 SD Did Not Alter Locomotor Activity and Anxiety‐Like Behavior in WT but Improved the Deficits in 3xTg‐AD Mice

**FIGURE 2:**
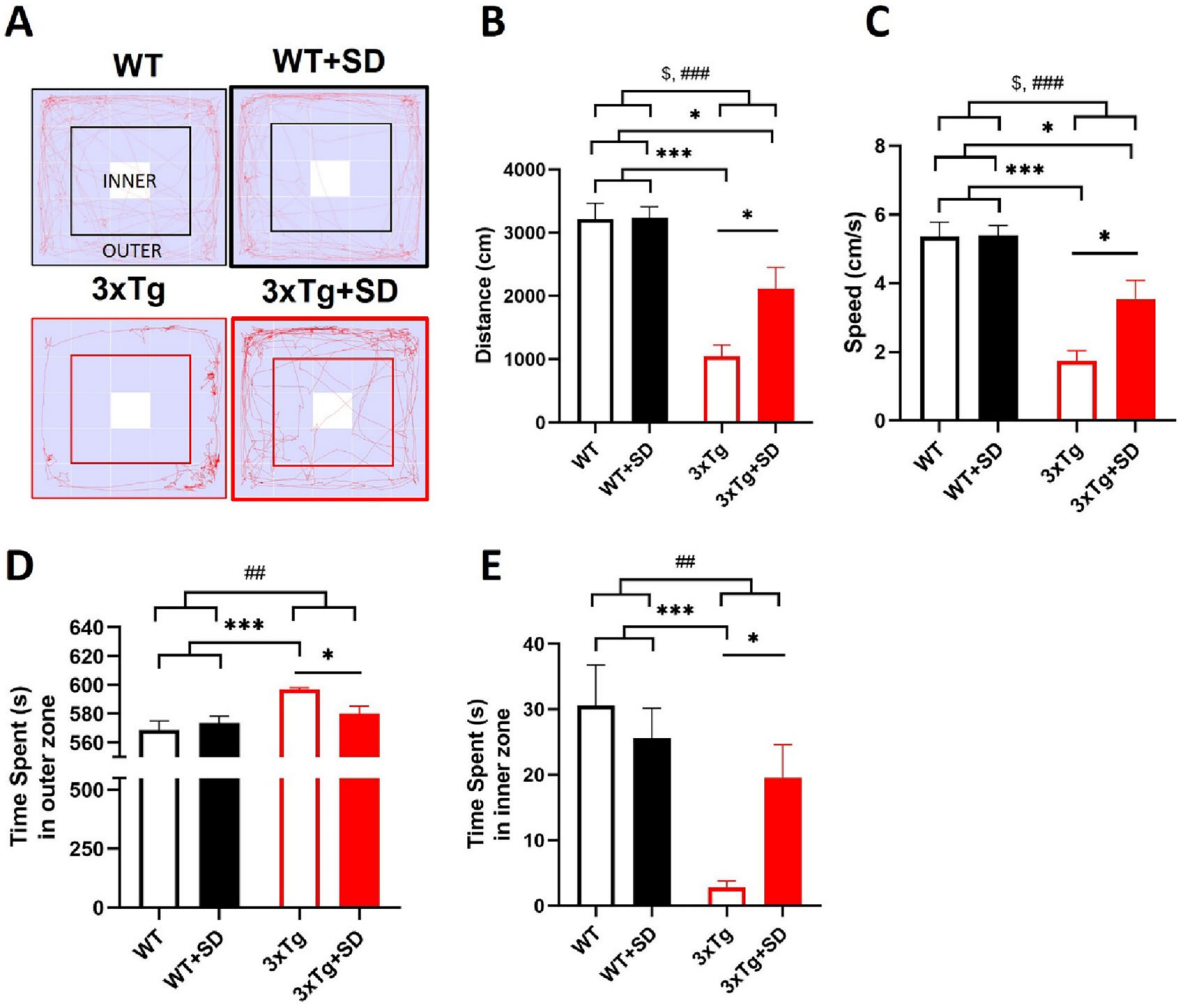
**SD did not alter locomotor activity and anxiety‐like behavior of the WT but improved the deficits in 3xTg‐AD mice. (A)** The tracing diagram of mice in the OFT, **(B)** The distance traveled, **(C)** The speed of WT and 3xTg‐AD mice with/without SD induction, and the time spent in **(D)** The outer, and (E) inner zones by each group of mice. All the results were plotted as Mean±SEM, and statistical differences were calculated for the interaction of two factors (genotypes and SD induction) by two‐way ANOVA and among groups by the Mann–Whitney *U* test. ^*^Indicated *p*‐value < 0.05 and ^***^Indicated *p*‐value < 0.001 when compared among groups. ^##^Indicated *p*‐value < 0.01 and ^###^Indicated *p*‐value < 0.001 when compared between genotypes. ^$^Indicated *p*‐value < 0.05 when compared between SD induction. WT (*n* = 13), WT+SD (*n* = 10), 3xTg (*n* = 8), 3xTg+SD (*n* = 9).

To investigate the effect of SD on the locomotor activity and anxiety‐like behavior of 3xTg‐AD mice, we have performed an OFT. The tracing diagram of mice for 10 min is shown in Figure 2A. The results demonstrated that genotypes of mice affected the SD induction in distance traveled (*F*_(1, 40)_ = 4.351, *p* < 0.044) and speed (*F*_(1, 40)_ = 4.363, *p* < 0.044). The WT+SD mice have no significant difference in locomotor activity compared to the WT mice. The 3xTg mice demonstrated less distance traveled (*p* < 0.001; Figure 2B) and speed (*p* < 0.001; Figure 2C) than the WT and WT+SD mice. In addition, the distance traveled (*p* < 0.001) and speed (*p* < 0.001) of the 3xTg+SD mice were also significantly reduced compared to the WT and WT+SD mice. Notably, the 3xTg+SD mice showed a significant increase in speed (*p* < 0.036) and distance traveled (*p* < 0.036) compared to the 3xTg mice.

To determine anxiety‐like behavior using OFT, the time spent by mice in the inner and outer zones of the chamber was calculated. The statistical analysis revealed that the SD induction was affected by the genotypes of mice in terms of time spent in the inner (*F*_(1, 40)_ = 4.282, *p* < 0.046) and outer zones (*F*_(1, 40)_ = 4.143, *p* < 0.049). The WT+SD mice performed similarly in anxiety‐like behavior compared to the WT mice. The 3xTg mice that stayed in the outer zone (Figure 2D) were significantly more compared to the WT (*p* < 0.001) and WT+SD (*p* < 0.001) mice while significantly less compared to the 3xTg+SD (*p* < 0.021) mice. On the other hand, the 3xTg mice spent significantly less time staying in the inner zone (Figure 2E) compared to the WT (*p* < 0.001) and WT+SD (*p* < 0.001) mice but more time compared to the 3xTg+SD (*p* < 0.015) mice.

### 3.3 SD Did Not Affect Novel Object Recognition of the WT but Helped Enhance NOR Performance of 3xTg‐AD Mice

**FIGURE 3:**
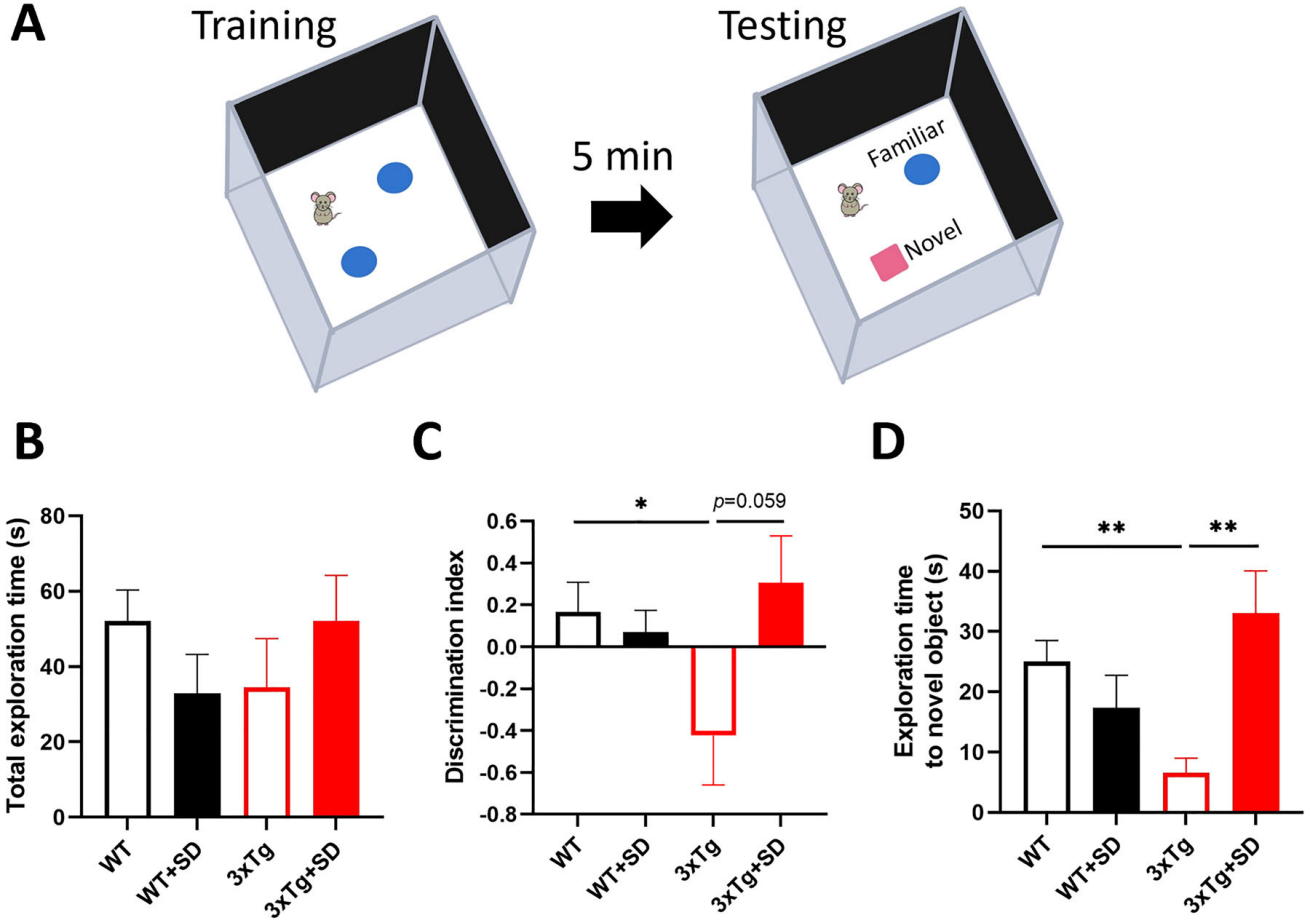
**SD did not affect novel object recognition of the WT but helped enhance NOR performance of 3xTg‐AD mice. (A)** The experimental protocol for the novel object recognition (NOR) test, **(B)** The total time the mice spent exploring familiar and novel objects, **(C)** The discrimination index for all groups (plus value = toward a novel object and minus value = toward a familiar object), and **(D)** The exploration time to novel object of each group of mice. All the results were plotted as Mean±SEM, and statistical differences were calculated for the interaction of two factors (genotypes and SD induction) by two‐way ANOVA and among groups by the Mann–Whitney *U* test. **Indicated *p*‐value < 0.01 when compared among groups. WT (*n* = 13), WT+SD (*n* = 10), 3xTg (*n* = 8), 3xTg+SD (*n* = 9).

To clarify the impact of SD on the performance of non‐spatial WM or objective recognition memory, we carried out the NOR test. As shown in Figure 3A, the mice were trained with two similar objects, followed by five‐minute intervals and testing the familiar and novel objects. There was no statistical difference in the interaction between the genotype and the SD induction on total exploration time (*F*_(3, 40)_ = 2.975, *p* = 0.093) but significantly different in discrimination index (*F*_(3, 40)_ = 5.541, *p* < 0.024) and exploration time to the novel object (*F*_(3, 40)_ = 12.154, *p* < 0.001). The total exportation time of mice among groups showed no significant differences (Figure 3B). The discrimination index (Figure 3C) of the WT+SD (*p* = 0.522) and 3xTg+SD (*p* = 0.431) mice showed no significant differences. In contrast, the 3xTg (*p* < 0.045) mice were significantly decreased compared to WT mice. Remarkably, the 3xTg+SD (*p* = 0.059) mice appeared to increase in the discrimination index compared to the 3xTg mice. Considering the exploration time for the novel object (Figure 3D), the 3xTg mice spent significantly less time than the WT mice (*p* < 0.003), while the 3xTg+SD mice spent significantly more time than the 3xTg mice (*p* < 0.004). However, the WT+SD mice have no significant differences in the exploration time of the novel object compared to the WT mice (*p* = 0.284). It suggested that SD does not impair non‐spatial WM in the WT+SD mice but helps improve non‐spatial WM deficits in the 3xTg‐AD mice.

### 3.4 The Expression of GABAergic Signaling Molecules in Different Brain Areas After SD

#### 3.4.1 Prefrontal Cortex

The PFC is strongly related to the SD and working memory (Verweij et al. 2014); however, the expression of GABAergic signaling in this brain area during REM‐SD in the 3xTg‐AD mice has not been investigated yet. Therefore, we conducted immunohistochemistry and found that the interaction between the genotypes and SD induction was statistically significant for the expression of the GABA (*F*_(3, 35)_ = 5.771, *p* < 0.022), GAD67 (*F*_(3, 36)_ = 10.024, *p* < 0.003), and GABA_B_R1 receptor (*F*_(3, 36)_ = 20.271, *p* < 0.001) in the PFC but not the GABA_A_α1 receptor (*F*_(3, 36)_ = 1.369, *p* = 0.251).

**FIGURE 4:**
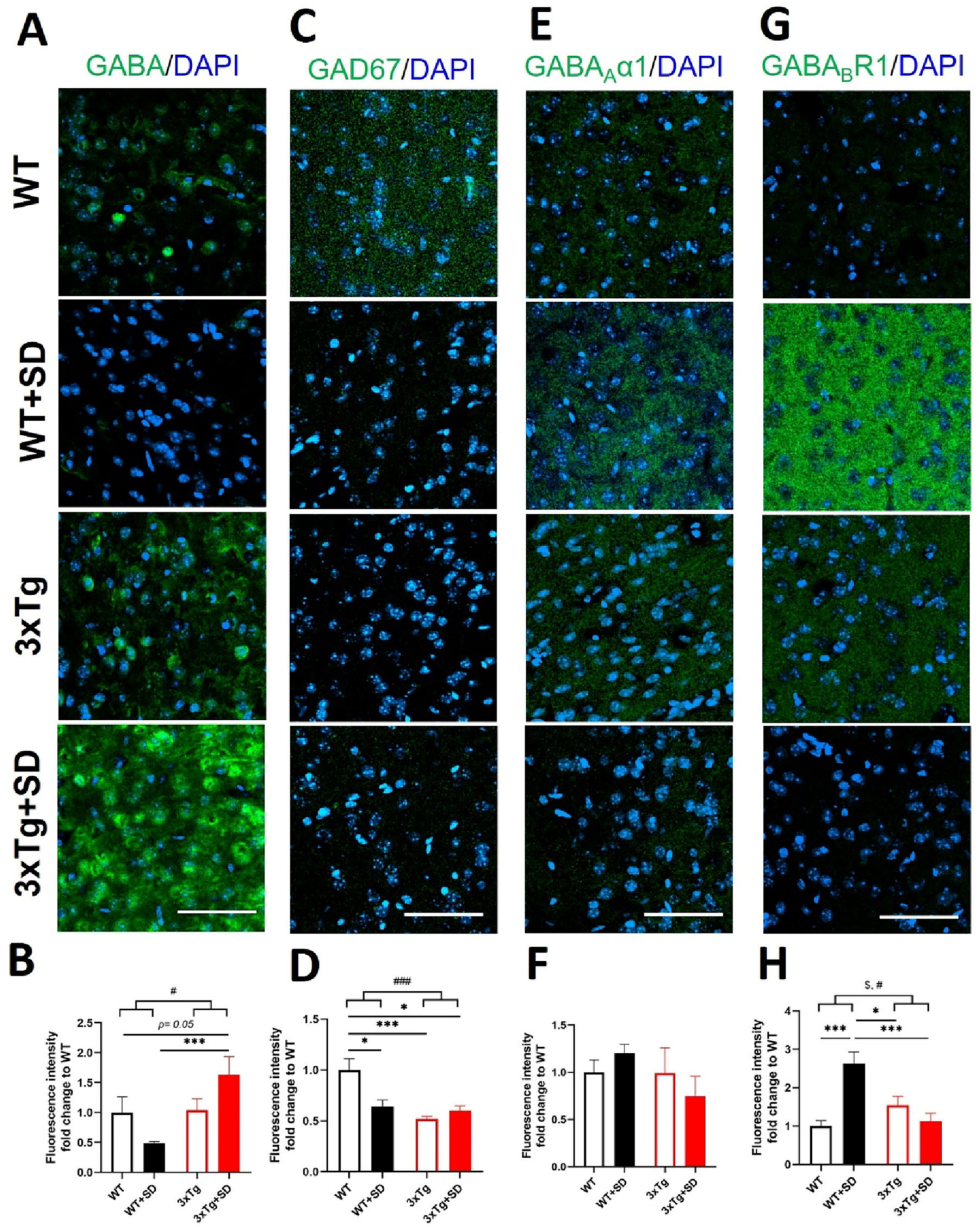
**The expression of GABA‐related molecules in the prefrontal cortex after SD**. Immunofluorescence staining and the quantitative results of **(A, B)** GABA, **(C, D)** GAD67, **(E, F)** GABA _A_α1, and **(G, H)** GABA_B_R1 in the prefrontal cortex of the WT and 3xTg‐AD mice after SD. All the results were plotted as Mean±SEM, and statistical differences were calculated for the interaction of two factors (genotypes and SD induction) by two‐way ANOVA and among groups by the Mann–Whitney *U* test. ^*^Indicated *p*‐value < 0.05, and ^***^Indicated *p*‐value < 0.001 when compared among groups. ^#^Indicated *p*‐value < 0.05 and ^###^Indicated *p*‐value < 0.001 when compared between genotypes. ^$^Indicated *p*‐value < 0.05 when compared between SD induction. GABA‐related molecules (green) and DAPI (blue; nuclei). Bar = 100 µm and n = 3 /group.

The GABA levels were similar for WT and 3xTg mice (*p* = 0.796). The GABA levels in the WT+SD mice appear to decline compared to WT mice (*p* = 0.200). It is worth noting that the GABA levels in the 3xTg+SD mice increased compared to the 3xTg (*p* = 0.387) and WT mice (*p* = 0.050) but were significantly different compared to WT+SD mice (*p* < 0.001; Figure 4A, B). In addition, we found lower expression of GAD67 in the PFC (Figure 4C, D) of the WT+SD (*p* < 0.019), 3xTg (*p* < 0.001), and 3xTg+SD (*p* < 0.014) mice than the WT mice. No significant changes were observed in the expression of the GABA_A_α1 receptor among groups in the PFC (Figure 4E, F). Remarkably, the GABA_B_R1 receptor expression (*p* < 0.001; Figure 6A, B) in the WT+SD mice was significantly increased compared to the WT mice. In addition, the positive signal levels of the GABA_B_R1 receptor in the PFC of the WT+SD mice were considerably greater than that of the 3xTg (*p* < 0.019) and 3xTg+SD (*p* < 0.001) mice.

#### 3.4.2 Hippocampal CA1 Area

We next determined the expression of GABAergic signaling in the hippocampal CA1 area. The hippocampus is a well‐studied brain area linked with learning and memory and is susceptible to SD (Spanò et al. 2020). The CA1 subregion is known to be responsible for memory consolidation, late retrieval, and recognition (Mueller et al. 2011). The interaction between the genotype and SD induction was significantly different in the expression levels of GABA (*F*_(3, 33)_ = 4.738, *p* < 0.038), GABA_B_R1 receptor (*F*_(3, 36)_ = 5.878, *p* < 0.021), and GAD 67 (*F*_(3, 36)_ = 9.883, *p* < 0.004) but no difference in GABA_A_α1 receptor (*F*_(3, 36)_ = 0.002, *p* = 0.962) expression.

**FIGURE 5:**
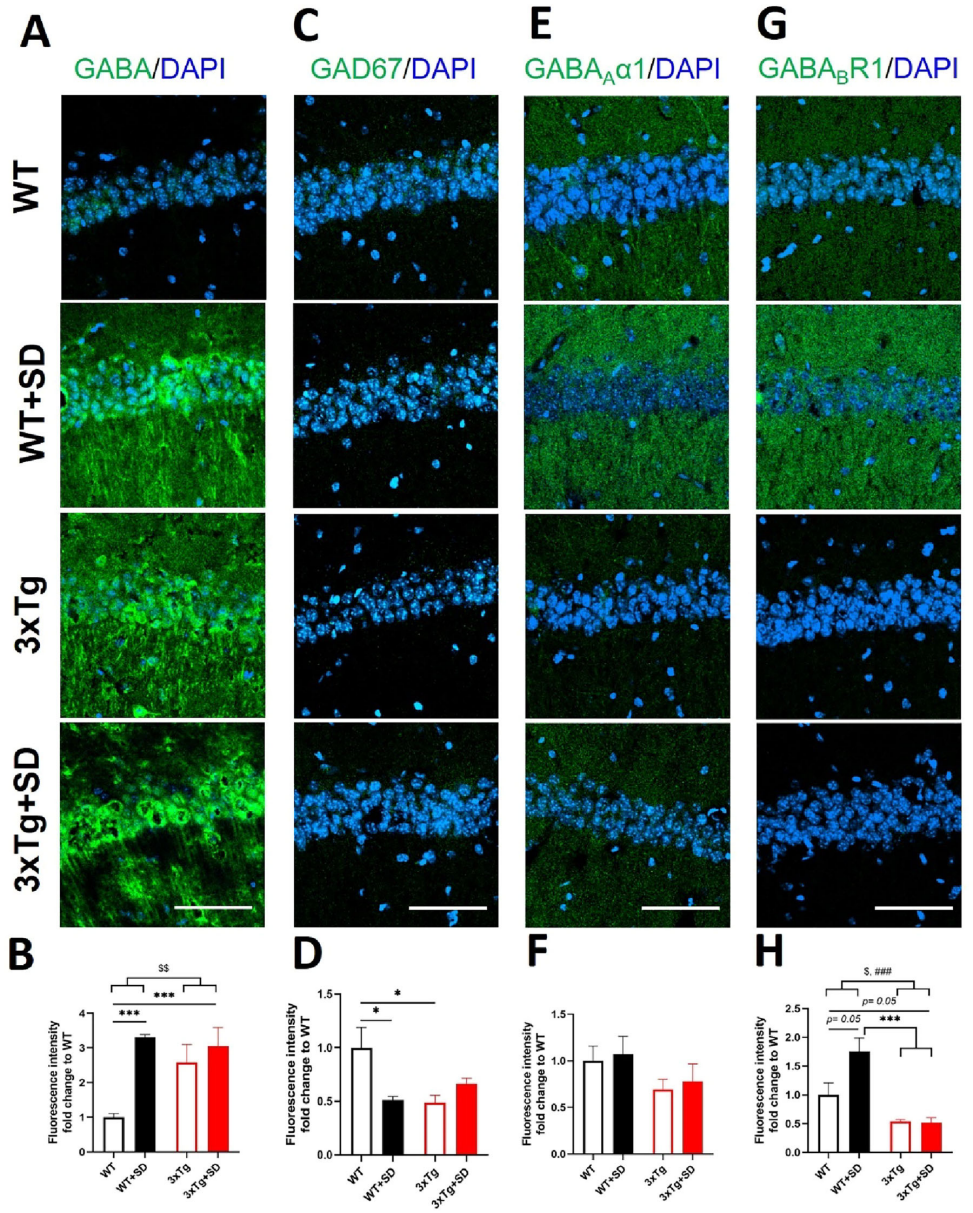
**The expression of GABA‐related molecules in the hippocampal CA1 area after SD**. Immunofluorescence staining and the quantitative results of **(A, B)** GABA, **(C, D)** GAD67, **(E, F)** GABA _A_α1, and **(G, H)** GABA_B_R1 in the hippocampal CA1 area of the WT and 3xTg‐AD mice after SD. All the results were plotted as Mean±SEM, and statistical differences were calculated for the interaction of two factors (genotypes and SD induction) by two‐way ANOVA and among groups by the Mann–Whitney *U* test. ^*^Indicated *p*‐value < 0.05, and ^***^Indicated *p*‐value < 0.001 when compared among groups. ^###^Indicated *p*‐value < 0.001 when compared between genotypes. ^$^Indicated *p*‐value < 0.05 and ^$$^Indicated *p*‐value < 0.01 when compared between SD induction. GABA‐related molecules (green) and DAPI (blue; nuclei). Bar = 100 µm and *n* = 3/group.

The GABA expression level in the hippocampal CA1 region (Figure 5A, B) of the WT+SD (*p* < 0.001) and 3xTg+SD (*p* < 0.001) mice were significantly enhanced compared to WT mice. The GABA expression level of the 3xTg mice appeared to be higher than the WT mice (*p* = 0.094). On the other hand, the positive signals of the GAD67 (Figure 5C, D) were significantly reduced in the hippocampal CA1 area of the WT+SD (*p* < 0.011) and 3xTg (*p* < 0.024) mice compared to the WT mice. Meanwhile, we did not observe a significant change in the expression of the GABA_A_α1 receptor among groups (Figure 5E, F). Notably, the expression of the GABA_B_R1 receptor in the WT+SD mice appeared to be increased compared to the WT mice (*p* = 0.050; Figure 5G, H) and was significantly higher than those in the 3xTg (*p* < 0.001) and 3xTg+SD (*p* < 0.001) mice.

#### 3.4.3 Hippocampal CA3 Area

The hippocampal CA3 subregion is important for memory encoding and early retrieval (Mueller et al. 2011). The results of immunofluorescent staining in the CA3 subregion revealed significant alterations in the expression of the GABA_B_R1 receptor (*F*_(3, 35)_ = 60.005, *p* < 0.001) but not GABA (*F*_(3, 33)_ = 0.055, *p* = 0.817), GAD67 (*F*_(3, 36)_ = 2.921, *p* = 0.097), and GABA_A_α1 receptor (*F*_(3, 36)_ = 3.660, *p* = 0.065) across different genotypes and under SD conditions.

**FIGURE 6:**
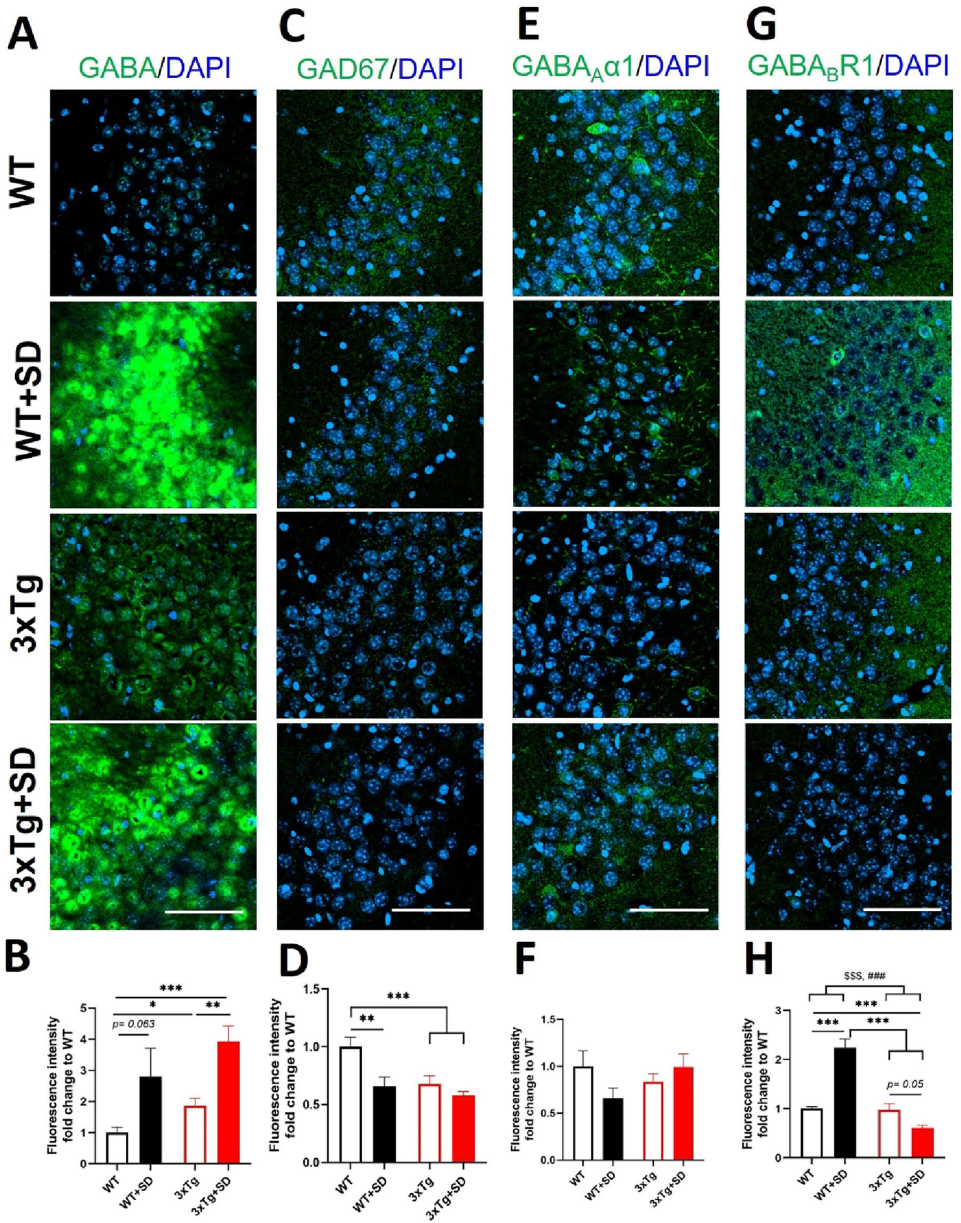
**The expression of GABA‐related molecules in the hippocampal CA3 area after SD**. Immunofluorescence staining and the quantitative results of **(A, B)** GABA, **(C, D)** GAD67, **(E, F)** GABA _A_α1, and **(G, H)** GABA_B_R1 in the hippocampal CA3 area of the WT and 3xTg‐AD mice after SD. All the results were plotted as Mean±SEM, and statistical differences were calculated for the interaction of two factors (genotypes and SD induction) by two‐way ANOVA and among groups by Mann–Whitney *U* test. ^*^Indicated *p*‐value < 0.05, ^**^Indicated *p*‐value < 0.01, and ^***^Indicated *p*‐value < 0.001 when compared among groups. ^###^Indicated *p*‐value < 0.001 when compared between genotypes. ^$$$^Indicated *p*‐value < 0.001 when compared between SD induction. GABA‐related molecules (green) and DAPI (blue; nuclei). Bar = 100 µm and *n* = 3/group.

The levels of GABA in the hippocampal CA3 area (Figure 6A, B) were found to be elevated (*p* = 0.063) in the WT+SD mice and considerably increased in the 3xTg (*p* < 0.012) and 3xTg+SD (*p* < 0.001) mice compared to the WT mice. Notably, the GABA levels in the hippocampal CA3 region of the 3xTg+SD mice were significantly higher than the 3xTg mice (*p* < 0.002). On the other hand, the WT+SD (*p* < 0.008), 3xTg (*p* < 0.014), and 3xTg+SD (*p* < 0.001) mice exhibited a decline in the GAD67 expression (Figure 6C, D) compared to the WT mice. No significant changes were noted in the expression of the GABA_A_α1 receptor among groups in the hippocampal CA3 subregion (Figure 6E, F). Besides, the expression of the GABA_B_R1 receptor in the WT+SD mice was significantly increased compared to the WT mice (*p* < 0.001; Figure 6G, H) and was higher than those in the 3xTg (*p* < 0.001) and 3xTg+SD (*p* < 0.001) mice. The GABA_B_R1 receptor expression in the hippocampus CA3 area of the 3xTg+SD mice showed a significant decrease compared to the WT mice (*p* < 0.001) but appeared to be reduced compared to the 3xTg mice (p = 0.050).

#### 3.4.4 Dentate Gyrus

Next, we measured the expression of GABAergic signaling in the DG, the hippocampal sub‐region that is important for sleep‐dependent memory consolidation and encoding (Spanò et al. 2020). We found that the interaction between the genotypes and SD induction was statistically significant for the expression of the GABA_B_R1 receptor (*F*_(3, 36)_ = 20.238, *p* < 0.001) but not GABA (*F*_(3, 34)_ = 0.313, *p* = 0.580), GAD67 (*F*_(3, 34)_ = 3.687, *p* = 0.064, and GABA_A_α1 receptor (*F*_(3, 35)_ = 2.296, *p* = 0.140) in the DG.

**FIGURE 7:**
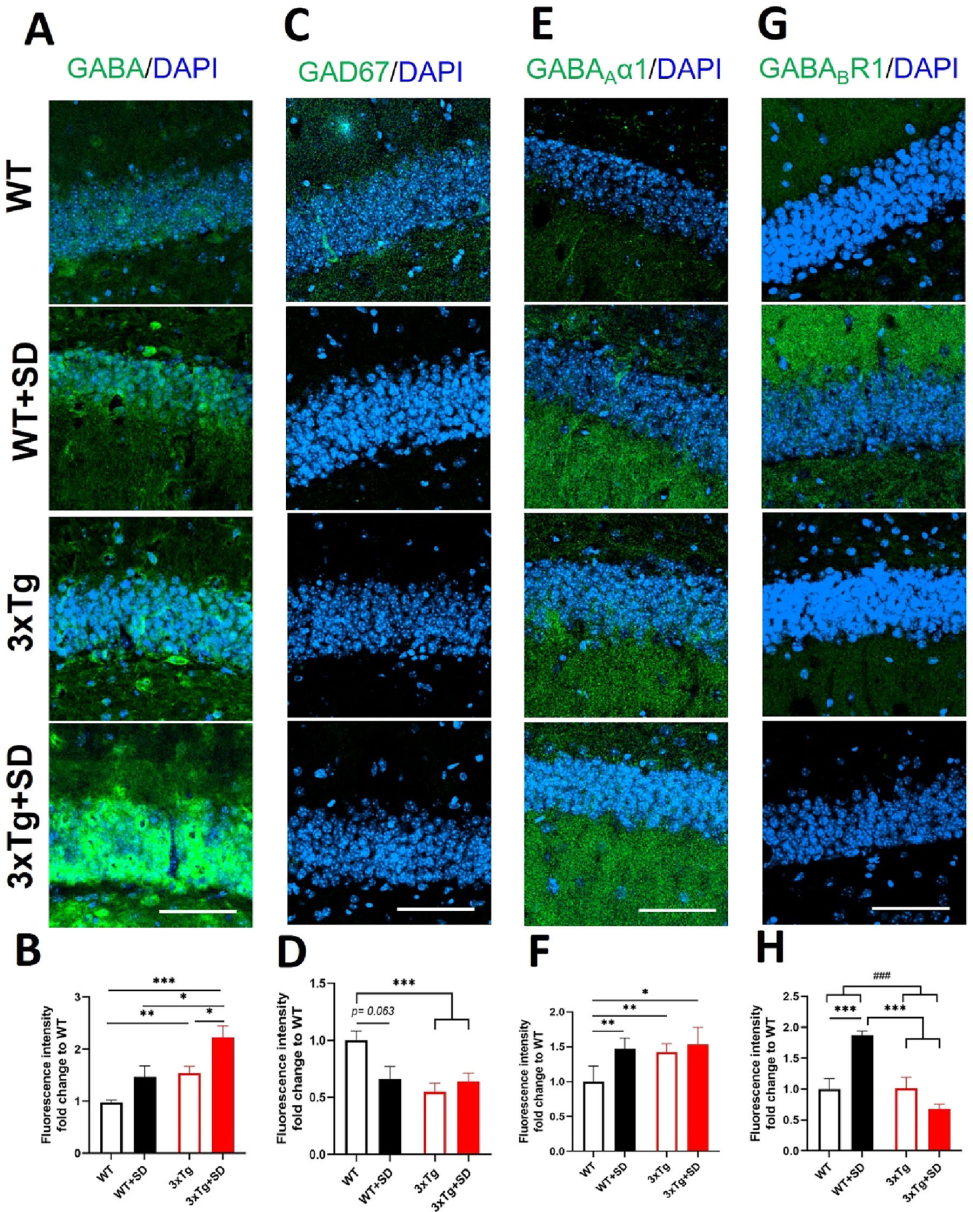
**The expression of GABA‐related molecules in the dentate gyrus after SD**. Immunofluorescence staining and the quantitative results of **(A**, **B)** GABA, **(C**, **D)** GAD67, **(E, F)** GABA _A_α1, and **(G, H)** GABA_B_R1 in the hippocampal CA1 area of the WT and 3xTg‐AD mice after SD. All the results were plotted as Mean±SEM, and statistical differences were calculated for the interaction of two factors (genotypes and SD induction) by two‐way ANOVA and among groups by the Mann–Whitney *U* test. ^*^Indicated *p*‐value < 0.05, ^**^Indicated *p*‐value < 0.01, and ^***^Indicated *p*‐value < 0.001 when compared among groups. ^###^Indicated *p*‐value < 0.001 when compared between genotypes. GABA‐related molecules (green) and DAPI (blue; nuclei). Bar = 100 µm and *n* = 3/group.

The GABA levels (Figure 7A, B) in the DG of the 3xTg (*p* < 0.008) and 3xTg+SD (*p* < 0.001) mice were significantly higher compared to WT mice and appeared to rise in WT+SD mice (*p* = 0.114). In addition, the GABA levels in the 3xTg+SD mice were significantly higher than the 3xTg mice (*p* < 0.024) and WT+SD mice (*p* < 0.019). Conversely, the expression of GAD67 (Figure 7C, D) decreased considerably in the 3xTg (*p* < 0.001) and 3xTg+SD mice (*p* < 0.001) and appeared to reduce in the WT+SD mice (*p* = 0.063) compared to the WT mice. At the same time, the expression of the GABA_A_α1 receptor (Figure 7E, F) in the DG of the WT+SD (*p* < 0.006), 3xTg (*p* < 0.008), and 3xTg+SD (*p* < 0.027) mice was considerably higher than that in the WT mice. The expression of the GABA_B_R1 receptor in the DG (*p* < 0.001; Figure 7G, H) of the WT+SD mice was significantly increased compared to the WT mice and was considerably greater than that of the 3xTg (*p* < 0.001) and 3xTg+SD (*p* < 0.001) mice.

### 3.5 The Correlations Between GABA‐related Molecule Expression and Behavior Performance

**FIGURE 8:**
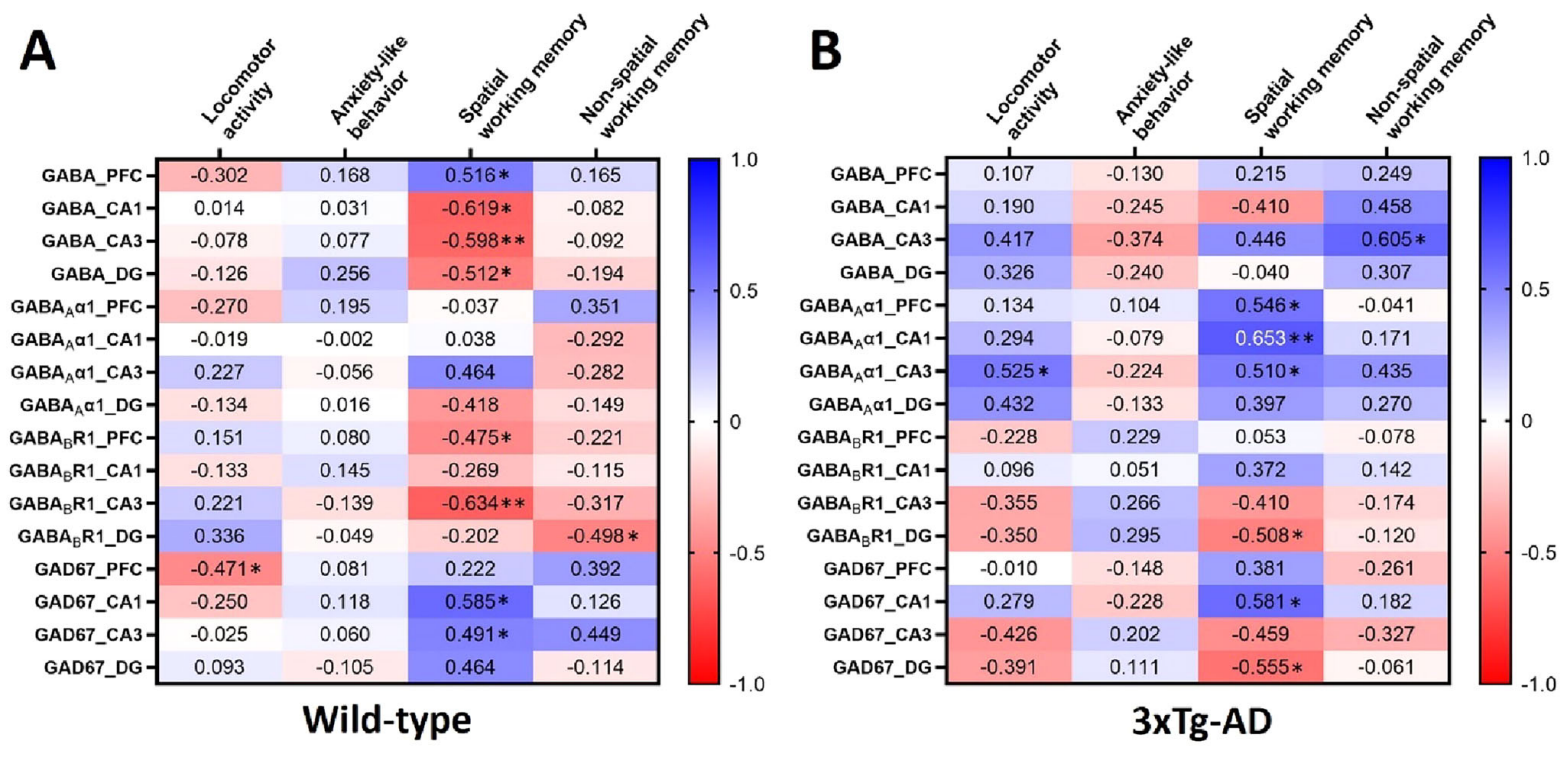
**The correlations between GABAergic signaling expression in different brain areas and behavior performances of the WT and 3xTg‐AD mice**. The figure demonstrated the correlation between the behavior performance, including locomotor activity, anxiety‐like behavior, spatial, and non‐spatial working memory, and the expression of GABA, GABA_A_α1, GABA _B_R1, and GAD67 in the prefrontal cortex (PFA) and hippocampal regions CA1, CA3, and DG of the **(A)** WT and **(B)** 3xTg‐AD mice. The positive correlation is represented by blue, while the negative correlation is represented by red. The correlation (two‐sided) is *indicated *p*‐value < 0.05 and **indicated *p*‐value < 0.01.

We further conducted a Pearson correlation coefficient analysis to understand whether the GABAergic signaling changes were associated with behavior outcomes of the WT (Figure 8A) and 3xTg‐AD (Figure 8B) mice, including locomotor activity (Supplementary Table 1), anxiety‐like behavior (Supplementary Table 2), spatial (Supplementary Table 3), and non‐spatial WM (Supplementary Table 4). In the WT mice, locomotor activity was negatively correlated with the GAD67 expression in the PFC (*r =* ‐0.471*, p* < 0.049). In contrast, locomotory activity of the 3xTg‐AD mice was positively correlated with the expression of the GABA_A_α1 receptor in the hippocampal regions CA3 (*r =* 0.525*, p* < 0.030). For the anxiety‐like behavior, we found that there was no significant correlation with GABAergic signaling changes in both WT and 3xTg‐AD mice.

Remarkably, the spatial WM performance of the WT mice was positively correlated with the GABA levels in the PFC (*r =* 0.516*, p* < 0.034) and the expression of GAD67 in the hippocampal subregion CA1 (*r =* 0.585*, p* < 0.011) and CA3 (*r =* 0.491*, p* < 0.039). We also noted a negative correlation with the GABA levels in the hippocampal subregions CA1 (*r = ‐*0.619*, p* < 0.014), CA3 (*r = ‐*0.598*, p* < 0.009), and DG (*r = ‐*0.512*, p* < 0.043). The expression of the GABA_B_R1 receptor in the PFC (*r = ‐*0.475*, p* < 0.046) and hippocampal CA3 subregion (*r = ‐*0.634*, p* < 0.006) also appear in a negative correlation. In the 3xTg‐AD mice, the spatial WM performance was positively correlated with the expression of the GAD67 in the hippocampal CA1 area (*r =* 0.581*, p* < 0.015) and the GABA_A_α1 receptor in the PFC (*r =* 0.546*, p* < 0.023) hippocampal subregions CA1(*r =* 0.653*, p* < 0.004) and CA3 (*r =* 0.510*, p* < 0.036). On the other hand, the spatial WM performance of the 3xTg‐AD mice demonstrates a negative correlation with the expression of the GABA_B_R1 receptor in the PFC (*r = ‐*0.508*, p* < 0.037) and the GAD67 in the DG of the hippocampus (*r = ‐*0.555*, p* < 0.032).

Our study found a negative correlation between the non‐spatial WM performance of the WT mice and the expression of the GABA_B_R1 receptor in the DG of the hippocampus. In contrast, we found a positive correlation between the non‐spatial WM performance of 3xTg‐AD mice and the GABA levels in the hippocampal CA3 subregion. These results delineate relationships between the changes of GABA‐related molecules and behavior performance under SD influence, particularly spatial WM.

## 4 Discussion

The present study reveals the impact of REM‐SD on GABAergic signaling and WM performance in WT and 3xTg‐AD mice. Our findings indicate that 6‐month‐old 3xTg‐AD mice exhibit impairments in locomotor activity, anxiety‐like behavior, and both spatial and non‐spatial WM. REM‐SD attenuated deficits in locomotor activity, anxiety‐like behavior, and non‐spatial WM in 3xTg‐AD mice without affecting spatial WM. In contrast, REM‐SD impaired spatial WM in WT mice, leaving other behaviors unaffected. Additionally, REM‐SD‐induced changes in GABA_A_α1, GABA_B_R1, and GAD67 expression were region‐ and genotype‐specific, with distinct patterns observed between WT and 3xTg‐AD mice. These results highlight significant interactions between genotype and SD that influence behavior and GABAergic signaling.

REM‐SD has complicated effects on locomotor activity and anxiety‐like behaviors, with outcomes depending on deprivation duration, methodology, and animal models. Studies using short‐term REM‐SD (24 h) in rats reported no significant changes in locomotor activity (Mahdavi et al. 2021; Xie et al. 2018) and anxiety‐like behaviors (Turan et al. 2021) that were similar to behaviors of the WT+SD mice in our study. In contrast, Zhang K et al. reported that 72 h of REM‐SD decreased locomotion in mice (Zhang et al. 2017). Unexpectedly, we found that REM‐SD improved impairments in locomotion and anxiety‐like behaviors of the 3xTg‐AD mice (Kim et al. 2022; Martinez‐Gonzalez et al. 2004); however, the insight mechanisms related to this phenomenon need further investigation.

In the NOR test for non‐spatial WM, REM‐SD did not affect the NOR performance of WT mice, as also reported by Nurhan Enginar's group (Ozakman et al. 2021). We demonstrated that REM‐SD surprisingly improved non‐spatial WM deficits in 3xTg‐AD mice accompanied by enhanced GABA levels in the hippocampus, which supports Ghoshal A et al. reported that administering 0.5 to 1% of GABA to rats significantly increases the recognition index in the NOR test (Ghoshal and Conn 2015). This phenomenon may result from compensatory increases in neuroplasticity and neurotrophic factors like brain‐derived neurotrophic factor (BDNF) and vascular endothelial growth factor (Giacobbo et al. 2016; Gorgulu et al. 2022). Several studies reported that enhancing BDNF levels could promote object memory recognition performance by encouraging the growth and survival of neurons in the perirhinal cortex, an important brain area for the discrimination of overlapping object memories (Callaghan and Kelly Á 2012; Hopkins and Bucci 2010). These findings suggest that increasing neurotrophic factors and GABA levels after REM‐SD may ameliorate non‐spatial WM impairment in the 3xTg‐AD mice.

We also found that REM‐SD impaired WM performance in WT mice, which supports previous studies that SD for 36 h could affect spatial WM in young men aged 21 to 28 (Peng et al. 2020) and 12 h of SD could induce spatial WM deficits in mice (Hagewoud et al. 2010). Spatial WM was also impaired in 3xTg‐AD mice, in line with previous experiments tested in 8‐arm radial mazes (Clark et al. 2015a) and T‐mazes (Suresh et al. 2022) demonstrating that SD deteriorates WM in 3xTg‐AD mice. These findings suggest that REM‐SD may increase AD risk in healthy animals but does not worsen spatial WM deficits in 3xTg‐AD mice, likely because their cognitive function is already maximally impaired.

The PFC and hippocampus are crucial brain regions for executing various types of memory, including episodic memory (Eichenbaum 2017), recognition memory (C. Wang et al. 2021), and spatial WM (Spellman et al. 2015). The PFC‐hippocampal pathway projects primarily to excitatory pyramidal cells and GABAergic interneurons (Ghoshal and Conn 2015), suggesting that alterations in GABAergic signaling within these regions could significantly impact memory performance.

Our study found that alterations in GABA‐related molecules in the PFC and hippocampus possibly correlate to locomotor activity but not anxiety‐like behavior in the WT and 3xTg‐AD mice. The low GAD67 and high GABA_A_α1 expressions observed after REM‐SD in the WT and 3xTg‐AD mice are associated with increasing locomotor activity. A prior study reported that mice lacking GAD67 in parvalbumin neurons (Fujihara et al. 2015) and global knockdown GAD67 mice could present hyperlocomotion in the OFT (Miyata et al. 2021). In addition, mice showed enhanced locomotor activity when receiving zolpidem (3 mg/kg), a selective GABA_A_α1 receptor agonist, in repeated doses but not for a single dosage (Vlainic and Pericic 2009).

The correlation patterns of GABA levels in the PFC with WM performance after REM‐SD differed between WT and 3xTg‐AD mice. A previous human study by Ragland J. D. et al. found that GABA levels were linked to WM performance but had different effects in healthy subjects versus schizophrenia patients (Ragland et al. 2020). Our study recorded decreased GABA levels in the PFC of WT mice but an increase in 3xTg‐AD mice after REM‐SD, with both genotypes showing WM deficits. It suggests that optimal GABA levels are crucial for WM performance, which provides a direction for developing therapeutic targets to attenuate spatial dysfunction often observed in early AD.

Furthermore, increased GABA_B_R1 expression in the PFC and hippocampus of WT mice after REM‐SD was associated with impaired spatial WM, consistent with studies showing that GABA_B_ receptor agonists can impair spatial memory (Stackman and Walsh 1994), while GABA_B_ receptor antagonist administration improved spatial memory deficits (Helm et al. 2005; Song et al. 2021). Conversely, decreased GABA_A_α1 receptor expression in the PFC and hippocampal CA1 region correlated with poor spatial WM performance in 3xTg‐AD mice, highlighting the importance of GABA_A_α1 receptors in maintaining spatial WM in AD (Soto et al. 2013; Taherianfard and Taci 2015).

Lesion studies have shown that different subregions of the hippocampus are involved in spatial WM. Lesions in the hippocampal CA1 area of mice have been associated with spatial WM impairments, as assessed by the Y‐maze test (Dillon et al. 2008). Similarly, lesions in the hippocampal CA3 region have been linked to deficits in forming and recalling spatial WM (Jo et al. 2007). Our study found that poor spatial WM performance was correlated with decreased GAD67 levels in the hippocampal CA1 and CA3 regions of WT mice and the CA1 region of 3xTg‐AD mice following REM‐SD. These findings are consistent with previous studies on GAD67 knockout mice, which also showed spatial reference and WM deficits (Fujihara et al. 2020). In the TgCRND8 AD mouse model, decreased GAD67 levels in the CA1 and CA3 regions were associated with AD pathologies (Krantic et al. 2012). These results suggest that GAD67, which mediates GABA synthesis in the hippocampal CA1 and CA3 regions, plays a crucial role in spatial WM performance after REM‐SD. Collectively, these findings underscore a critical association between spatial WM performance and the GABAergic signaling pathway, implicating this mechanism in the pathophysiology of REM‐SD‐induced cognitive impairments in both WT and AD mouse models.

## 5 Conclusion

In conclusion, the present study demonstrates that REM sleep deprivation elicits genotype‐specific alterations in GABAergic signaling within the prefrontal cortex and hippocampus, which are associated with distinct impairments in working memory performance. These findings contribute to a more nuanced understanding of the intricate interactions among sleep regulation, neurotransmitter dynamics, and cognitive function in both physiological conditions and AD‐affected brains.

## Author Contributions

**Peeraporn Varinthra**: conceptualization, investigation, methodology, formal analysis, writing – original draft, writing – review and editing. **Shu‐Ching Shih**: methodology, investigation. **Ingrid Y Liu**: writing–review and editing, supervision, resources, funding acquisition, conceptualization, project administration, validation, visualization.

## Conflicts of Interest

The authors declare that the research was carried out without commercial or financial associations, which may be considered a possible conflict of interest.

### Peer Review

The peer review history for this article is available at https://publons.com/publon/10.1002/brb3.70607.

## Supporting information



Supporting Information

## Data Availability

The data used during the study are available from the corresponding authors by request.
