# Supplementary material for: GABAergic Signaling Underlying REM Sleep Deprivation‐Induced Spatial Working Memory Deficits
Source: Brain Behav. 2025 Jun 2;15(6):e70607. doi: 10.1002/brb3.70607 (PMC12127666; doi:10.1002/brb3.70607)
Supplement: Supplementary file 1 — Supporting Information [file BRB3-15-e70607-s001.docx]

**Supplementary Table**

**Supplementary Table 1** Results of Pearson correlation coefficient (*r*) analysis based on locomotor activity

| **Parameters** | **Locomotor activity (Distance traveled)** | | | |
| --- | --- | --- | --- | --- |
|  | **WT** | | **3xTg** | |
|  | ***r*** | ***P*-value** | ***r*** | ***P*-value** |
| GABA_PFC | -0.302 | 0.239 | 0.107 | 0.684 |
| GABA_CA1 | 0.014 | 0.959 | 0.190 | 0.465 |
| GABA_CA3 | -0.078 | 0.758 | 0.417 | 0.138 |
| GABA_DG | -0.126 | 0.643 | 0.326 | 0.201 |
| GABA_A_α1_PFC | -0.270 | 0.278 | 0.134 | 0.607 |
| GABA_A_α1_CA1 | -0.019 | 0.939 | 0.294 | 0.252 |
| GABA_A_α1_CA3 | 0.227 | 0.366 | 0.525* | 0.030 |
| GABA_A_α1_DG | -0.134 | 0.609 | 0.432 | 0.083 |
| GABA_B_R1_PFC | 0.151 | 0.551 | -0.228 | 0.380 |
| GABA _B_R1_CA1 | -0.133 | 0.598 | 0.096 | 0.713 |
| GABA _B_R1_CA3 | 0.221 | 0.393 | -0.355 | 0.162 |
| GABA _B_R1_DG | 0.336 | 0.173 | -0.350 | 0.169 |
| GAD67_PFC | -0.471* | 0.049 | -0.010 | 0.969 |
| GAD67_CA1 | -0.250 | 0.317 | 0.279 | 0.277 |
| GAD67_CA3 | -0.025 | 0.922 | -0.426 | 0.089 |
| GAD67_DG | 0.093 | 0.714 | -0.391 | 0.149 |

The correlation (two-sided) is * Indicated p-value < 0.05, PFC = prefrontal cortex, DG = dentate gyrus of the hippocampus

**Supplementary Table 2** Results of Pearson correlation coefficient (*r*) analysis based on anxiety-like behavior

| **Parameters** | **Anxiety-like behavior (Time spent in the outer zone)** | | | |
| --- | --- | --- | --- | --- |
|  | **WT** | | **3xTg** | |
|  | ***r*** | ***P*-value** | ***r*** | ***P*-value** |
| GABA_PFC | 0.168 | 0.519 | -0.130 | 0.619 |
| GABA_CA1 | 0.031 | 0.912 | -0.245 | 0.342 |
| GABA_CA3 | 0.077 | 0.760 | -0.374 | 0.187 |
| GABA_DG | 0.256 | 0.338 | -0.240 | 0.353 |
| GABA_A_α1_PFC | 0.195 | 0.439 | 0.104 | 0.692 |
| GABA_A_α1_CA1 | -0.002 | 0.995 | -0.079 | 0.762 |
| GABA_A_α1_CA3 | -0.056 | 0.824 | -0.224 | 0.388 |
| GABA_A_α1_DG | 0.016 | 0.953 | -0.133 | 0.611 |
| GABA_B_R1_PFC | 0.080 | 0.752 | 0.229 | 0.376 |
| GABA _B_R1_CA1 | 0.145 | 0.566 | 0.051 | 0.846 |
| GABA _B_R1_CA3 | -0.139 | 0.595 | 0.266 | 0.302 |
| GABA _B_R1_DG | -0.049 | 0.847 | 0.295 | 0.250 |
| GAD67_PFC | 0.081 | 0.751 | -0.148 | 0.571 |
| GAD67_CA1 | 0.118 | 0.642 | -0.228 | 0.379 |
| GAD67_CA3 | 0.060 | 0.812 | 0.202 | 0.436 |
| GAD67_DG | -0.105 | 0.679 | 0.111 | 0.693 |

PFC = prefrontal cortex, DG = dentate gyrus of the hippocampus

**Supplementary Table 3** Results of Pearson correlation coefficient (*r*) analysis based on spatial working memory performance

| **Parameters** | **Spatial working memory (Alternation %)** | | | |
| --- | --- | --- | --- | --- |
|  | **WT** | | **3xTg** | |
|  | ***r*** | ***P*-value** | ***r*** | ***P*-value** |
| GABA_PFC | 0.516* | 0.034 | 0.215 | 0.406 |
| GABA_CA1 | -0.619* | 0.014 | -0.410 | 0.102 |
| GABA_CA3 | -0.598** | 0.009 | 0.446 | 0.110 |
| GABA_DG | -0.512* | 0.043 | -0.040 | 0.880 |
| GABA_A_α1_PFC | -0.037 | 0.884 | 0.546* | 0.023 |
| GABA_A_α1_CA1 | 0.038 | 0.880 | 0.653** | 0.004 |
| GABA_A_α1_CA3 | 0.464 | 0.052 | 0.510* | 0.036 |
| GABA_A_α1_DG | -0.418 | 0.095 | 0.397 | 0.114 |
| GABA_B_R1_PFC | -0.475* | 0.046 | 0.053 | 0.839 |
| GABA _B_R1_CA1 | -0.269 | 0.281 | 0.372 | 0.142 |
| GABA _B_R1_CA3 | -0.634** | 0.006 | -0.410 | 0.102 |
| GABA _B_R1_DG | -0.202 | 0.422 | -0.508* | 0.037 |
| GAD67_PFC | 0.222 | 0.376 | 0.381 | 0.132 |
| GAD67_CA1 | 0.585* | 0.011 | 0.581* | 0.015 |
| GAD67_CA3 | 0.491* | 0.039 | -0.459 | 0.064 |
| GAD67_DG | 0.464 | 0.052 | -0.555* | 0.032 |

The correlation (two-sided) is * Indicated p-value < 0.05 and ** indicated p-value < 0.01., PFC = prefrontal cortex, DG = dentate gyrus of the hippocampus

**Supplementary Table 4** Results of Pearson correlation coefficient (*r*) analysis based on non-spatial working memory performance

| **Parameters** | **Non-spatial working memory (Discrimination Index)** | | | |
| --- | --- | --- | --- | --- |
|  | **WT** | | **3xTg** | |
|  | ***r*** | ***P*-value** | ***r*** | ***P*-value** |
| GABA_PFC | 0.165 | 0.526 | 0.249 | 0.335 |
| GABA_CA1 | -0.082 | 0.772 | 0.458 | 0.064 |
| GABA_CA3 | -0.092 | 0.717 | 0.605* | 0.022 |
| GABA_DG | -0.194 | 0.471 | 0.307 | 0.231 |
| GABA_A_α1_PFC | 0.351 | 0.153 | -0.041 | 0.875 |
| GABA_A_α1_CA1 | -0.292 | 0.240 | 0.171 | 0.512 |
| GABA_A_α1_CA3 | -0.282 | 0.257 | 0.435 | 0.081 |
| GABA_A_α1_DG | -0.149 | 0.567 | 0.270 | 0.294 |
| GABA_B_R1_PFC | -0.221 | 0.377 | -0.078 | 0.766 |
| GABA _B_R1_CA1 | -0.115 | 0.651 | 0.142 | 0.587 |
| GABA _B_R1_CA3 | -0.317 | 0.215 | -0.174 | 0.504 |
| GABA _B_R1_DG | -0.498* | 0.035 | -0.120 | 0.646 |
| GAD67_PFC | 0.392 | 0.108 | -0.261 | 0.312 |
| GAD67_CA1 | 0.126 | 0.618 | 0.182 | 0.484 |
| GAD67_CA3 | 0.449 | 0.061 | -0.327 | 0.200 |
| GAD67_DG | -0.114 | 0.654 | -0.061 | 0.829 |

The correlation (two-sided) is * Indicated p-value < 0.05, PFC = prefrontal cortex, DG = dentate gyrus of the hippocampus
